# Cross-sectional study on the drug utilization and evaluation indicator of antibiotics used in pediatric population

**DOI:** 10.1186/s12913-021-06727-3

**Published:** 2021-10-13

**Authors:** Xu Hu, Xueting Zhang, Yao Wang, Xuefeng Xie

**Affiliations:** 1 Department of Basic and Clinical Pharmacology, Anhui Institute of Innovative Drugs, School of Pharmacy, Anhui Medical University, 81 Meishan Road, Hefei 230032 Anhui, People’s Republic of China; 2 Inflammation and Immune-Mediated Diseases Laboratory of Anhui Province, Hefei, People’s Republic of China

**Keywords:** Defined daily dose, Prescription daily dose, Paediatric prescription, Antibacterial drugs, Drug utilisation

## Abstract

**Background:**

The lack of medication standards is a serious problem in paediatrics mainly because of age-related differences in organ development and physiological functions in children. Consequently, dosage measurement becomes inaccurate. For this reason, methods for evaluating and monitoring rational paediatric medications should be developed. Drug use indicators, such as those similar to the drug utilisation index (DUI) based on the Anatomical Therapeutic Chemical/Defined Daily Dose (DDD) and widely used for the assessment of appropriate dosage in adults, should be explored in terms of their applicability to children.

**Methods:**

A total of 5,538 prescriptions of antibiotics selected from a general teaching hospital were included. Drug, dose, frequency and treatment duration were obtained from each prescription. The prescription daily dose (PDD) of each antibiotic drug was calculated as the average of the daily doses. Underdose and overdose were determined in terms of the PDD/DDD ratio for each prescription. Children’s DUI (cDUI) was explored in terms of the appropriate dosage for children as follows: the meaning of children’s DDD (cDDD) and the evaluation of paediatric drug dosage.

**Results:**

The top five antibiotics and their utilisation rates were as follows: cefmetazole sodium injection (18.47 %), erythromycin lactobionate injection (15.07 %), amoxicillin/clavulanate potassium injection (10.72 %), ceftriaxone sodium injection (9.50 %) and azithromycin dry suspension (8.02 %). The ratio of cDUI and PDD/cDDD was mostly not close to 1.

**Conclusions:**

The establishment of a cDUI system is an effective means of paediatric dosage evaluation. In addition to DDDs, cDUI and PDD/cDDD should be used to analyse the utilisation of antibiotics in children.

## Background

The irrational use of antibiotics is a public health problem and has an impact on people’s health and economic development [1–3]. In the 1990 s, many countries, such as the United States, launched antimicrobial stewardship programmes [4, 5] to promote the rational use of antibiotics.

Having a low immune function, children are prone to infectious diseases. As such, they have emerged as one of the groups that widely use antibiotics, and their medication safety has remarkably concerned the society. Paediatric dosage cannot be directly normalised on the basis of adult dosage [6] because the exposure of antibiotics largely differs between paediatric patients and adults [7].

In contrast to adults who are mature in all aspects, in a healthy paediatric population, age directly affects the development status and physiological functions of organs; consequently, paediatric drug use cannot be accurately measured, off-label drug use behaviour [8, 9] is inevitable, and a series of drug safety problems in serious cases occurs [10]. The medication error rate without injury amongst paediatric patients is three times higher than that amongst adults [11], and the medication error rates of causing injury and death in the former are 31 and 13 % higher than those in the latter [12]. Therefore, evaluating the rational use of antibiotics in paediatrics is a difficult problem. The index of drug intensity based on a limited daily dose is recommended by the World Health Organisation (WHO) and the health administration department of China. However, the limited daily dose has restrictions in the children’s population. For this reason, studies should explore how to scientifically and accurately understand the current situation of the use of antibiotics in children and reasonably evaluate the clinical use of antibiotics.

This study analysed the utilisation of antibiotics in paediatric inpatients in the comprehensive teaching hospital of Anhui Medical University as an example and the indicators of paediatric drug utilisation index (DUI) and prescription daily dose (PDD) in the clinical comprehensive evaluation of paediatric antibiotics to protect children’s health rights and interests, avoid the risk of drug use and improve the rationality and safety of antibiotic use in paediatrics. This study also aimed to establish a methodology for the evaluation of antimicrobial agents in children and provide a reference for rational clinical drug use.

## Data and methods

### Sources

In this study, the comprehensive teaching hospital of Anhui Medical University was selected as the sample point by convenience sampling to collect all the prescriptions of antibiotic treatment for children hospitalised from 1 to 2019 to 31 May 2020 via the hospital information system. The inclusion criteria were the prescriptions for patients aged 0–14 years [13–15] and the exclusion criteria were the prescriptions with missing information or obviously wrong data. Inconsistencies with the complete prescription definition in the *Prescription Management Measures* and inaccuracies in the writing of the data on review by the pharmacist are considered to be data errors or omissions [16].

### Drug use research

To compare drug use at a general level, the WHO has adopted Anatomical Therapeutic Chemical (ATC) and defined daily dose (DDD) system [17] referred to as the ATC/DDD system. DDD is defined as the average daily dose of adults used for therapeutic purposes [18]. The main indicators commonly used in drug utilisation research and rational drug use evaluation includes the following based on the DDD method: DDDs, DUI and PDD [18–20]. DUI represents the trend of drug use and can be used as a criterion to judge whether clinical drug use is reasonable. PDD is the actual daily dose of the drug at the time of prescription. The combination of DUI and PDD was helpful to explain the specific details of drug utilization.

$$ \mathrm{DDDs}=\mathrm{drug}\ \mathrm{consumption}\ \left(\mathrm{g}\right)/\mathrm{DDD} $$

$$ \mathrm{DUI}=\mathrm{DDDs}/\mathrm{actual}\ \mathrm{medication}\ \mathrm{days} $$

The WHO and related studies have concluded that prescription drug use is basically reasonable when DUI is close to 1. Considering the influence of other factors during the course of medication, this study regarded DUI as close to 1 when it was between 0.9 and 1.1 [21–23]. DUI > 1.1 indicates that the actual daily dose of a given drug exceeds DDD and implies drug abuse. Conversely, DUI < 0.9 denotes that the actual daily dose of this drug is lower than DDD and may affect the therapeutic effect because of the excessively low dose. Of course, DUI is mainly used in clinical practice to monitor drug utilization trends, and a more accurate dose needs to be determined by clinicians and pharmacists after diagnosis and discussion.

Paediatric patients were the main object of this study. Indicators such as children’s defined daily dose (cDDD) and children’s DUI (cDUI) were used to distinguish adult dose and calculated using the same formula described above. The dosage of paediatric drugs is directly related to age, so the children were divided into five age groups in accordance with the *New Edition of Pharmacology* (17th edition) and the child growth standards of the WHO [14, 22]. Each age group had the corresponding cDDD combined with the actual prescription situation of hospitals: newborns (1–28 days): cDDD = 1/10 − 1/8DDD; infants (28 days-1 year): cDDD = 1/8 − 1/4 DDD; toddlers (1–3 years): CDDD = 1/4 − 1/3DDD; preschool (4–5 years): cDDD = 1/3 − 1/2DDD; school age (6–14 years): cDDD = 1/2–2/3DDD [14].

In *New Edition of Pharmacology*, a body surface area method is introduced to calculate dose, but the exact dose for clinical use needs to be determined by clinician’s diagnosis and clinical pharmacist’s evaluation. The calculation of body weight and gestational age are also introduced in this book, but these are factors that have been comprehensively considered by clinical pharmacists in the process of prescription review and this study is not authorized to further review, so we temporarily do not calculate these two factors.

### Rationality evaluation

In accordance with *Chinese Pharmacopoeia* (2015 Edition), *Guidelines for Clinical Application of Antimicrobial Agents* (2015 Edition), *China National Formulary* [24, 25] and package inserts, the rationality evaluation standard of paediatric antibacterial drug prescription was established, whether the use of antibacterial drugs met the relevant specifications was evaluated, and the medication characteristics of irrational prescriptions were analysed. A prescription is considered reasonable when it meets all the criteria. The rationality evaluation standard of paediatric antibacterial drug prescription was as follows: 1.there should be obvious symptoms; 2.the appropriate drug should be selected according to according to the symptoms; 3.the dosage should be adjusted according to different ages; 4.the dosage form and route should be selected according to the drug and patient compliance; 5.there should be no incompatibility or adverse reactions(Due to the route of administration, dosage form and dosage and other reasons).

### Statistical analysis

The prescriptions were sorted out in Excel 2010 software. Factors related to patient age, drug class, infection type, dosage form, specification, price and time of administration were included, and the baseline data table of patients was drawn for the corresponding calculation. Statistical analysis was conducted in SPSS 23.0 software. Differences were considered clinically significant when *P* < 0.05.

## Results

### Basic information

After the data were screened, 5,538 prescriptions that met the inclusion and exclusion criteria were collected. After classification, the baseline data of the patients were obtained. The average age of the patients was 2.72 ± 3.35 years, the average hospitalisation time was 8.96 ± 9.60 days, and the average medication time was 2.97 ± 2.95 days (Table 1).

**Table 1:** Baseline data of patients (*n* = 5538)

Variable	Number (n)	Proportion (%)
**Gender**		
male	3287	59.35
female	2251	40.65
**Age**		
Newborn	1458	26.33
Baby	781	14.10
Toddler	1712	30.91
Preschool	570	10.29
School age	1017	18.37
**Department**		
Respiratory blood endocrine	1913	34.54
Newborn	1564	28.25
Kidney rheumatism digestion cardiovascular	1457	26.31
Neurorehabilitation	451	8.14
ICU	153	2.76
**Length of stay**		
0~10	4411	79.65
11~20	766	13.83
21~30	141	2.55
31~60	185	3.34
>60	35	0.63
**Type of infection**		
Lower respiratory tract	3849	41.60
Blood	1693	18.30
Upper respiratory tract	1147	12.39
Gastrointestinal tract	799	8.63
Other	755	8.16
Central nervous system	369	3.99
Skin	254	2.74
Cardiovascular system	159	1.72
Urinary tract	144	1.56
Oral	84	0.91
**Irrational prescriptions**		
No obvious indications	674	72.16
Contraindications or adverse reactions	149	15.95
Unsuitable drug selection	95	10.17
Improper usage and dosage	12	1.29
Improper dosage form or route	4	0.43

Note: The ICU stands for Intensive Care Unit

### Types of infection

We double-checked the admission and discharge diagnoses of patients to confirm the type and number of infections in each patient, and counted them by person. Amongst the 5,538 prescriptions, 10 kinds of infections were involved, and some patients had multiple infections, with a total of 9,253 infections. Upper respiratory tract, lower respiratory tract and blood infections were the common types, accounting for 72.30 % (Table 1).

### Rationality evaluation of initial prescription antibiotics

According to the *Guidelines for Clinical Application of Antimicrobial Agents* (2015 Edition) and *New Edition of Pharmacology* (17th edition), the rationality of the sample prescriptions was preliminarily evaluated by more than two pharmacists in charge. According to the *Prescription Management Measures* issued by the Health Commission, PRC, a three-level quality control mode for prescription comment was established. Level 1 quality control is completed by the dispensing pharmacist, who evaluates the legality and normative suitability of the prescription. Secondary quality control was completed by multiple clinical pharmacists. The prescriptions were evaluated by referring to prescription evaluation standards, and the results of primary quality control were verified. Clinical pharmacists are independent of each other and do not interfere with each other. Finally, the results are summarized. Third-level quality control is performed by the pharmacy department and the pharmaceutical board, mainly to review the prescriptions that have differences in the second-level quality control. Of the 5,538 prescriptions, 4,604 (83.13 %) were rational prescriptions, and 934 (16.87 %) were unreasonable prescriptions. The specific manifestations and composition of unreasonable prescriptions are shown in Table 1. Amongst the irrational prescriptions, the most common drug use without obvious indications accounted for 72.16 %(Table 1), which means that the use of antimicrobial drugs in the absence of clinical indications of infection or the scope of treatment of antimicrobial drugs inconsistent with clinical diagnosis.

### Research on drug utilisation trend

Amongst the 5,538 prescriptions, 41 kinds of antibiotics, namely, 18 kinds of nonrestricted antibiotics, 13 kinds of restricted antibiotics and 10 kinds of special antibiotics, were involved. Some prescriptions were used in combination. A total of 15,119 patients were treated with antimicrobial agents. The top 20 antibacterial drug varieties in the prescription frequency of use and the corresponding DDD [26] and PDD are presented in Table 2.

**Table 2:** Top 20 commonly used antibiotics in frequency (*n* = 15,119)

Name of drug	frequency	Proportion /%	DDD/g	PDD/g
Cefmetazole Sodium for Injection	2792	18.47	4	0.923 ± 0.582
Erythromycin Lactobionate for Injection	2279	15.07	1	0.315 ± 0.312
Amoxicillin/ Potassium Clavulanate for Injection	1621	10.72	3	0.644 ± 0.865
Ceftriaxone sodium for Injection	1437	9.50	2	1.038 ± 0.585
Azithromycin for Suspension	1213	8.02	0.3	0.133 ± 0.062
Cefoperazone / Sulbactam for Injection	1174	7.77	4	0.554 ± 0.785
Azithromycin Injection	651	4.31	0.5	0.236 ± 0.089
Cefdinir Dispersible Tablets	477	3.15	0.6	0.191 ± 0.094
Cefaclor Suspension	459	3.04	1	0.280 ± 0.134
Penicillin Sodium for Injection	442	2.92	3.6	0.315 ± 0.469
Cefamandole Nafate for Injection	388	2.57	6	1.010 ± 0.654
Meropenem for Injection	345	2.28	3	0.219 ± 0.394
Azithromycin Tablets	308	2.04	0.3	0.306 ± 0.108
Cefoxitin Sodium for Injection	236	1.56	6	1.489 ± 0.795
Fluconazole and Sodium Chloride Injection	233	1.54	0.2	0.028 ± 0.035
Amoxicillin / Clavulanate Potassium Tablets	192	1.27	1.5	0.621 ± 0.286
Erythromycin Enteric Capsules	129	0.85	1	0.642 ± 0.215
Benzathine Penicillin for Injection	125	0.83	3.6	0.803 ± 0.335
Ampicillin Sodium / Sulbactam Sodium for Injection	119	0.79	6	1.045 ± 1.148
Cefprozil Dispersible Tablets	118	0.78	1	0.346 ± 0.174
Other	381	2.52	-	-
Total	15,119	100	-	-

Note: PDD is the average daily dose of corresponding drugs [27, 28]

### Rationality evaluation of initial prescription antibiotics

Four factors, namely, patients’ gender, age, department and length of stay, were statistically analysed to determine the relationship between the patients’ basic information and the rationality of prescriptions (Table 3).

**Table 3:** Comparison of basic information of patients and rationality of prescription (α = 0.05)

Variate		Reasonable prescription	Unreasonable prescription	χ^**2**^	***P***	R
		number(%)	number(%)			
**Gender**	Male	2768(84.21%)	519(15.79%)	6.675	<0.05	-0.035
	Female	1836(81.56%)	415(18.44%)			
**Age**	Newborn	846(58.02%)	612(41.98%)	892.635	<0.05	0.303
	Baby	711(91.04%)	70(8.96%)			
	Toddler	1596(93.22%)	116(6.78%)			
	Preschool	521(91.40%)	49(8.60%)			
	School age	930(91.45%)	87(8.55%)			
**Department**	Newborn	919(58.76%)	645(41.24%)	1004.083	<0.05	0.380
	Neurorehabilitation	353(78.27%)	98(21.73%)			
	Respiratory blood endocrine	1830(95.66%)	83(4.34%)			
	Kidney rheumatism digestion cardiovascular	1364(93.62%)	93(6.38%)			
	ICU	138(90.20%)	15(9.80%)			
**Length of stay(d)**	0~10	3765(85.35%)	646(14.65%)	84.242	<0.05	-0.090
	11~20	568(74.15%)	198(25.85%)			
	21~30	101(71.63%)	40(28.37%)			
	31~60	138(74.59%)	47(25.41%)			
	>60	32(91.43%)	3(8.57%)			

The chi-square test revealed that gender, age, department and length of stay significantly differed (*P* < 0.05). The rationality of prescription might be related to gender, age, department and length of stay. However, the correlation coefficient between gender and length of stay was less than 0.1, suggesting that they were not the main influencing factors. Therefore, this study focused on the use of antibiotics in children with different ages.

### Drug utilisation analysis

In this study, the top five drugs were taken as an example to analyse the drug utilisation of children in different age groups. The main analysis indices were cDUI and PDD/cDDD [28] (PDD and cDDD are taken as the mean value in calculation). Similar to the criteria of cDUI, PDD/cDDD close to 1 indicated that the prescription dosage is basically appropriate, and the range from 0.9 to 1.1 could be regarded as close to 1. PDD/cDDD > 1.1 implied the possibility of excessive drug use. PDD/cDDD < 0.9 suggested an insufficient drug dosage.

### Cefmetazole sodium for injection

Drug utilisation amongst children treated with cefmetazole sodium injection was analysed on the basis of different age groups (Table 4). In the 5 age groups, cDUI and PDD/cDDD were basically less than 0.9, which indicated that cefmetazole sodium injection was underdosed in different age groups of paediatric patients.

### Erythromycin lactobionate for injection

Drug utilisation amongst children treated with erythromycin cefose injection according to different age groups (Table 4). Among the five age groups, the cDUI (taking the mean) and PDD/ CDDD of newborns were less than 1 and close to 1 in the other age groups. This result showed that the dosage of erythromycin cefaloctate injection was too low in newborns, but it was basically appropriate in other age groups.

### Amoxicillin/clavulanate potassium for injection

The use of amoxicillin/potassium clavulanate injection was analysed on the basis of different age groups (Table 4). In the 5 age groups, the cDUI and PDD/cDDD of newborns and infants were less than 0.9, the cDUI and PDD/cDDD of infants were close to 1, and the cDUI and PDD/cDDD of preschool and school-aged children were more than 1.1. These results indicated that amoxicillin/potassium clavulanic injection was underused in newborns and infants, appropriately used in infants and overdosed in preschool and school-aged children.

### Ceftriaxone Sodium for Injection

Drug utilization analysis was performed on children treated with ceftriaxone sodium for injection according to different age groups(Table 4). The cDUI and PDD/cDDD were all greater than 1.1, indicating the overdose of ceftriaxone sodium for injection in pediatric patients.

### Azithromycin dry suspension

Drug utilisation amongst children treated with azithromycin dry suspension was analysed on the basis of different age groups (Table 4). Amongst the 5 age groups, cDUI and PDD/cDDD of newborns were close to 1, whereas the cDUI and PDD/cDDD of the 4 other groups were greater than 1.1. This result showed that azithromycin dry suspension was used appropriately in newborns. In other age groups, this drug was overdosed.

**Table 4:** Drug utilization analysis in different age groups

	Frequency	Dosage / g	Medication time / d	PDD/g	cDDD/g	cDUI	PDD/cDDD
**Cefmetazole Sodium for Injection**							
Newborn	202	245.185	910	0.263±0.108	0.400~0.500	0.538~0.674	0.584
Baby	447	627.654	1056	0.545±0.244	0.500~1.000	0.594~1.189	0.727
Toddler	1396	2986.440	3184	0.837±0.348	1.000~1.333	0.704~0.938	0.717
Preschool	344	1119.450	808	1.247±0.529	1.333~2.000	0.520~0.693	0.748
School age	403	1822.200	935	1.670±0.683	2.000~2.667	0.365~0.974	0.716
**Erythromycin Lactobionate for Injection**							
Newborn	212	30.723	821	0.037±0.049	0.100~0.125	0.299~0.374	0.329
Baby	474	283.097	1403	0.172±0.082	0.125~0.250	0.807~1.614	0.917
Toddler	936	906.834	2602	0.291±0.122	0.250~0.333	1.046~1.394	0.998
Preschool	337	407.265	902	0.389±0.161	0.333~0.500	0.903~1.356	0.934
School age	320	599.263	908	0.584±0.253	0.500~0.667	0.989~1.320	1.001
**Moxicillin / Clavulanate Potassium for Injection**							
Newborn	1006	804.604	3551	0.166±0.067	0.300~0.375	0.604~0.755	0.492
Baby	129	154.282	369	0.399±0.319	0.375~0.750	0.557~1.115	0.709
Toddler	179	612.018	568	0.952±0.444	0.750~1.000	1.077~1.437	1.088
Preschool	62	371.410	210	1.637±0.508	1.000~1.500	1.179~1.769	1.310
School age	245	2266.690	952	2.202±0.829	1.500~2.000	1.190~1.587	1.258
**Ceftriaxone Sodium for Injection**							
Newborn	117	161.374	608	0.257±0.151	0.200~0.250	1.062~1.327	1.142
Baby	323	598.400	1099	0.561±0.203	0.250~0.500	1.089~2.178	1.496
Toddler	530	1436.530	1558	0.951±0.231	0.500~0.670	1.376~1.844	1.626
Preschool	183	702.680	505	1.361±0.283	0.670~1.000	1.391~2.077	1.630
School age	284	1801.530	926	1.813±0.391	1.000~1.333	1.459~1.945	1.554
**Azithromycin dry Suspension**							
Newborn	29	1.971	52	0.037±0.009	0.030~0.038	0.997~1.263	1.088
Baby	185	21.592	275	0.084±0.085	0.038~0.075	1.047~2.066	1.487
Toddler	660	113.414	924	0.124±0.041	0.075~0.100	1.227~1.637	1.417
Preschool	230	53.470	309	0.172±0.029	0.100~0.150	1.154~1.730	1.376
School age	109	26.914	126	0.212±0.054	0.150~0.200	1.068~1.424	1.211

### Clinical feasibility analysis

The dosages of different drugs used in the five age groups (based on the mean PDD) were compared via ANOVA(Table 5). The results showed that the doses significantly differed between the age group and the drug group (F1 = 10.419, *P*1 < 0.01; F2 = 9.319 and *P*2 < 0.01).

**Fig. 1:**
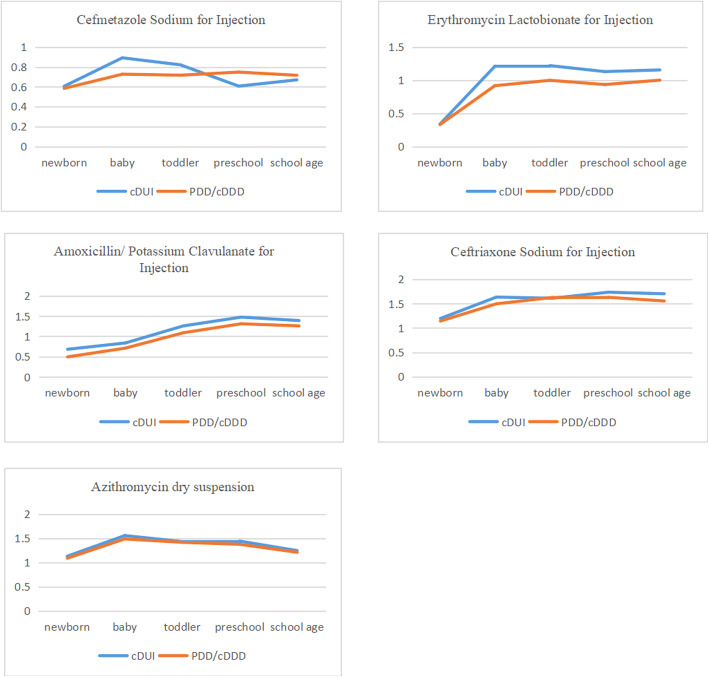
drug utilization trend of five drugs in different age groups

In Fig. 1, the drug utilisation trend of the five drugs in different age groups of paediatric patients was basically the same regardless of the analysis index used. Therefore, cDUI and PDD/cDDD could be preliminarily considered and used for the comprehensive evaluation of the clinical application of antibiotics in paediatrics. These indices also had clinical significance and feasibility for the analysis of paediatric drug utilisation.

**Table 5:** Comparison of dosage (g) of five antibiotics used in different age groups

Group	Cefmezole sodium for injection	Erythromycin lactose for injection	Amoxicillin/potassium clavulate for injection	Ceftriaxone sodium for injection	Azithromycin dry suspension	
Newborn	0.263	0.037	0.166	0.257	0.037	F1 = 10.419
Baby	0.545	0.172	0.399	0.561	0.084	*P*1 < 0.01
Toddler	0.837	0.291	0.952	0.951	0.124	F2 = 9.319
Preschool	1.247	0.389	1.637	1.361	0.172	*P*2 < 0.01
School age	1.670	0.584	2.202	1.813	0.212	

## Discussion

According to the WHO, DDD is the average daily dose for adult indications, so it cannot be directly used as the basis for prescribing children’s medication. In the absence of specific medication standards, some hospitals directly reduce the adult dose in half to obtain the children’s medication standard. However, this method has no scientific clinical basis, and it leads to the deviation of medication results. In the present study, cDDD, cDUI, PDD/cDDD and other indicators were introduced to analyse the drug use status amongst children by dividing them into different age groups and preliminarily establish a rational evaluation method of antimicrobial drug use in children.

### Use of antibiotics

In this study, the irrational prescriptions of antibiotics for children in the sample hospitals accounted for 16.87 %, which indicated that irrational drug use in medical institutions is relatively common. This phenomenon is attributed to two possible reasons. From the perspective of prescribing physicians, physicians may not have a comprehensive understanding of antibacterial drug use, so they tend to ignore the principles of the clinical application of antibacterial drugs, or physicians use antibiotics randomly and do not choose broad-spectrum antibacterial drugs in strict accordance with indications. Doctors’ attitude and knowledge level of medication are important factors affecting prescription behaviour [29–31]. Therefore, medical institutions should actively implement training on the antimicrobial knowledge of paediatricians, improve the level of drug use and require prescription physicians and reviewers to supervise one another to enhance the quality of paediatric prescriptions. From the perspective of patients receiving drugs, children are a special group, whose body functions develop and change with age, and doctors experience difficulty in mastering the metabolism and reactions of drugs in children. Coupled with the lack of standards for direct use in paediatric patients, deriving paediatric medications from adult doses is difficult and risky. Paediatric medicine is full of uncertainty in terms of individual treatments and lack of experience and standards to refer to. Furthermore, pharmacokinetic studies on paediatric patients should be strengthened by combining with the physiological characteristics of patients of different ages, formulating administration plans and strictly controlling drug indications and dosages.

According to prescription analysis, the top five antibiotics commonly used in paediatrics are cefmetazole sodium injection, erythromycin lactate injection, amoxicillin/potassium clavulanate injection, ceftriaxone sodium injection and azithromycin dry suspension, accounting for 61.78 % of the total antibacterial drugs. This result confirmed that injection remains the most commonly used route of administration in paediatrics because of its rapid and reliable action and its advantages of systemic or local localisation. However, the compliance of injections is poor, and direct intravenous injection has a high risk, so the principles of injection administration must be strictly observed. The five commonly used drugs are used to treat the most common upper and lower respiratory tract infections and blood infections in patients, but they are also prone to adverse reactions. With the complex physiological conditions of children, paediatric clinicians should cautiously prescribe such medications.

### Drug utilisation analysis

The indices of cefmetazole sodium injection and erythromycin lactobionate injection were less than or close to 1. The index of amoxicillin/clavulanate potassium injection was less than 1 in newborns and infants, close to 1 in infants and greater than 1 in preschool and school age. The index of ceftriaxone sodium injection and azithromycin dry suspension were basically greater than 1. This finding showed that doctors cautiously prescribed cefmetazole sodium injection at a small dosage. The use of erythromycin lactobionate injection was appropriate, and ceftriaxone sodium injection and azithromycin dry suspension were overdosed. Furthermore, the use of amoxicillin/clavulanate potassium injection fluctuated greatly, that is, it was increasingly administered with the age of patients increase the dosage. As for different ages, the utilisation index of 3 drugs in newborns was less than 1, and 2 drug use indices of less than 1 were observed in infants. The utilisation index of 2 drugs in toddlers was close to 1, the index of 3 drugs in preschool and school age was greater than 1. This finding indicated that physicians’ medication changed with patients’ age. In newborns and infants, medications were carefully used, and dosage was reduced. In toddlers, medications fluctuated reasonably. In preschool and school age of over 4 years, the overdose phenomenon appears. This observation might be due to the importance that doctors attach to children of different ages. In comparison with older children, children under 4 years of age have poor drug absorption and metabolism and have a higher probability of adverse reactions [32]. Therefore, physicians cautiously administer drugs.

## Limitations

This study has some limitations. Firstly, the study only selected a tertiary hospital with a single sample point. Secondly, the study focused on the analysis of drug use amongst children in different age groups but did not consider the effects of weight and combined medications, which might influence the results. Lastly, cDUI and PDD/cDDD are not commonly used to evaluate the appropriately of paediatric drug use, and the evaluation system needs further improvement and should be jointly discussed by more scholars.

## Data Availability

The datasets used and/or analyzed during the current study are available from the corresponding author on reasonable request.

## Abbreviations

WHO: World Health Organization
DUI: Drug Utilisation Index
DDD: Defined Daily Dose
DDDs: Defined Daily Doses
DDDs: Defined Daily Doses
cDDD: Children Defined Daily Dose
cDUI: Children Drug Utilisation Index
ATC: Anatomical Therapeutic Chemical
ASP: Antimicrobial Stewardship Program
